# Local anesthetic lidocaine decelerates cytoplasmic streaming in *Egeria densa* via altered actin dynamics and reactive oxygen species generation

**DOI:** 10.1080/15592324.2026.2726830

**Published:** 2026-09-16

**Authors:** Noriyasu Magari, Tomoko Kagenishi, Ken Yokawa

**Affiliations:** a Department of Anesthesiology, Keio University School of Medicine, Shinjuku, Tokyo, Japan; b Faculty of Engineering, Kitami Institute of Technology, Kitami, Hokkaido, Japan

**Keywords:** Cytoplasmic streaming, *Egeria densa*, local anesthetic, lidocaine, calcium influx, actin cytoskeleton, reactive oxygen species

## Abstract

Local anesthetics (LAs) block voltage-gated Na^+^ channels in mammals, but they also affect cytoplasmic streaming in plant cells. Although certain LAs inhibit streaming in algae such as *Chara*, the underlying mechanisms remain unclear. This study aimed to elucidate how LAs reduce cytoplasmic streaming velocity, focusing on the actomyosin system and oxidative stress responses, using lidocaine as a representative LA. Streaming velocity in *Egeria densa* leaves decreased reversibly after lidocaine treatment and recovered following a washout procedure. Confocal laser scanning microscopy of fluorescently stained actin filaments revealed that lidocaine disrupted F-actin structures. Electron spin resonance analysis showed that lidocaine induced reactive oxygen species (ROS) production in a dose-dependent manner, and spatiotemporal fluorescence analysis with ROS-sensitive probes demonstrated that ROS accumulated near the plasma membrane and increased over time; this process appeared to be mediated by nicotinamide adenine dinucleotide phosphate (NADPH) oxidases and Ca^2+^ influx. Application of antioxidants attenuated the lidocaine-induced reduction in streaming velocity. In conclusion, lidocaine slows cytoplasmic streaming in *E. densa* by eliciting a reversible, multifaceted response involving NADPH oxidase–derived ROS, Ca^2+^ influx, and actomyosin remodeling, offering mechanistic insight into the still poorly understood phenomenon of local anesthetic action in plants.

## 1. Introduction

In mammals, local anesthetics (LAs) produce analgesic effects by blocking voltage-gated Na^+^ channels (VGSCs) and thereby suppressing sensory neurotransmission. Due to their amphipathic nature,^1^ ^,^ ^2^ LAs readily cross the cell membrane in a lipophilic state and subsequently become ionized and hydrophilic within the cytoplasm, where they inhibit VGSCs from the intracellular side.^1^ Furthermore, incorporation of LAs into the lipid bilayer induces membrane expansion, alters membrane fluidity and organization, and consequently affects membrane protein function.^3^ ^,^ ^4^ In mouse fibroblasts, exposure to LAs was reported to reversibly induce cell swelling, while electron microscopy revealed marked reductions in microtubules and actin filaments as well as substantial loss of their membrane association.^5^ Collectively, these findings suggest that, beyond inhibition of their primary targets VGSCs, LAs exert secondary effects through alterations in the physical properties of cellular membranes and the cytoskeleton.

Cytoplasmic streaming is an active intracellular flow phenomenon observed in plant cells. Since its first description by Corti in 1774,^6^ algae and aquatic plants such as *Chara braunii* and *Egeria densa* have served as representative model organisms for studying cytoplasmic streaming because of their large cell size, optical transparency, and rapid streaming velocities. Cytoplasmic streaming is generally thought to arise from ATP-dependent transport of organelles by myosin XI along stable actin bundles.^7^ ^,^ ^8^ This intracellular streaming provides several important physiological advantages, including efficient transport of nutrients and carbon dioxide, regulation of cell size, and maintenance of organ orientation through gravity sensing.^9^ ^,^ ^10^

Several previous studies have shown that LAs inhibit cytoplasmic streaming in aquatic plants. Tetracaine irreversibly suppresses cytoplasmic streaming in *Chara australis* in a dose-dependent manner, and this inhibitory effect is significantly delayed by extracellular calcium.^11^ Lidocaine has likewise been reported to reversibly inhibit cytoplasmic streaming in *Vallisneria* cells.^12^ Those authors further demonstrated that the inhibitory effect was independent of the ionic composition of the extracellular medium, whereas elevated pH promoted intracellular entry of lidocaine, suggesting that intracellular structures such as the tonoplast may represent important targets. Although general anesthetics are known to disrupt the plant cytoskeleton through destabilization of microtubules and inhibition of intracellular transport,^13-15^ the effects of LAs on plant cytoskeletal organization remain largely unclear. Because cytoplasmic streaming depends on the actomyosin system, both structural disorganization and functional impairment of the cytoskeletal network are strongly implicated in the reduction or cessation of streaming velocity. However, direct visual evidence supporting this mechanism has not previously been reported. In the present study, we investigated how LAs inhibit cytoplasmic streaming, focusing particularly on cytoskeletal organization and the involvement of calcium ions. In addition, because LAs are known to induce reactive oxygen species (ROS) production in plant cells,^16^ ^,^ ^17^ we examined the relationship between lidocaine-induced ROS generation and cytoplasmic streaming.

## 2. Materials and methods

### 2.1. Culture conditions

*Egeria densa* plants were purchased from a local supply store (DCM Co., Ltd., Tokyo, Japan) and cultivated in tap water (Kitami City, Hokkaido, Japan; approximately 0.39 mM Na^+^, 0.05 mM K^+^, 0.20 mM Ca^2^⁺, and 0.08 mM Mg^2^⁺; pH 7.2). The plants were maintained at room temperature under a 16/8 h light/dark photoperiod with an LED light intensity of 30 μmol m^−2^ s^−1^ (PPFD).

### 2.2. Measurement of cytoplasmic streaming velocity

Leaves of *E. densa* were carefully excised at the leaf base and incubated for 30 min in tap water used for cultivation under bright light conditions (150 μmol m^−2^ s^−1^). Following incubation, the leaves were exposed for 30 min to cultivating water containing 1% (w/v) lidocaine, which serves as the standard concentration commonly used in this line of research, whereas cultivating water without lidocaine was used for the control treatment. After treatment, the leaves were rinsed twice with cultivating water before microscopic observation. Cytoplasmic streaming velocity was evaluated by tracking chloroplast movement. Videos of chloroplast movement were recorded using an Olympus BX51 microscope (Olympus, Tokyo, Japan) equipped with a 40× objective lens. Image acquisition was performed at 20 fps, and chloroplast trajectories were subsequently analyzed using the Manual Tracking plugin in Fiji (ImageJ 2.16.0/1.54p; www.fiji.sc). For each chloroplast, six positional coordinates were plotted at approximately 2-s intervals over a total observation period of 10 s. Streaming velocity was calculated by dividing total displacement by observation time. Five leaves were analyzed in each experiment, and five fields of view were selected from each leaf. Within each field, the five fastest-moving chloroplasts were chosen for analysis. Chloroplasts that moved out of focus or became difficult to track during imaging were excluded from the analysis.

To evaluate the involvement of oxidative stress, leaves were pretreated for 30 min immediately after harvesting with cultivating water containing 0.3 mM N-acetylcysteine (NAC), 2 mM ascorbic acid, or 10 μM VAS2870 supplemented with 0.1% dimethyl sulfoxide (DMSO). Following pretreatment, the leaves were rinsed twice with the tap water and subsequently exposed to the same lidocaine treatment described above.

For experiments examining recovery of cytoplasmic streaming velocity, leaves obtained from the same *E. densa* individual were used throughout the experimental series. Each leaf sequentially underwent three phases: pretreatment, treatment, and post-treatment. To evaluate whether lidocaine causes irreversible cytotoxicity, a higher concentration of 2% (w/v) lidocaine was applied during the treatment phase; verifying streaming recovery after exposure to this higher concentration ensures that the treatment induces no permanent cellular damage across all tested conditions. Except during microscopic observation, all incubation procedures were carried out at room temperature under continuous bright light (150 μmol m^−2^ s^−1^). During the pretreatment phase, leaves were incubated in culture water for 30 min before imaging. The same leaves were then transferred to culturing water containing 2% (w/v) lidocaine and incubated for an additional 30 min before imaging during the treatment phase. Subsequently, to verify the absence of irreversible cytotoxicity, the lidocaine-treated leaves were gently rinsed twice with the same cultivating water to remove remaining lidocaine without inducing mechanical stress, returned to cultivating water, and incubated for 60 min to allow recovery from the anesthetic before final imaging during the post-treatment phase. For each experimental condition, three fields of view were selected from each leaf, and three chloroplasts were analyzed within each field. A total of five leaves were examined, resulting in analysis of 45 chloroplasts for each experimental condition. Chloroplasts that became difficult to track were excluded from the analysis.

### 2.3. ESR measurement

*E. densa* plants were incubated overnight in Milli-Q water to minimize background noise during electron spin resonance (ESR) measurements, and leaves were excised immediately before each experiment. The leaves were placed in 6-well plates (five leaves per well) and treated collectively. A 250 μL volume of the culture fluid containing a spin-trapping agent with 40 μL/mL ethanol, 50 mM N-tert-butyl-*α*-(4-pyridyl-1-oxide) nitrone (POBN), and 10 mM potassium phosphate buffer (pH 6.0), with final lidocaine concentrations of 0, 0.1, 0.2, 0.5, 1, 2, 4, or 10% (w/v), was added to each well. Samples were then incubated quietly in the dark for 1 h at room temperature. After incubation, the culture fluid was collected into a flat quartz cell (LC12, JEOL Ltd., Tokyo, Japan) for ESR measurement. Experiments were performed using three biological replicates. Data from all replicates were averaged and presented as a single graph. ESR spectra were recorded using a JEOL JES-X320 spectrometer (JEOL Ltd., Tokyo, Japan) under the following conditions: magnetic field center, 335.5 ± 7.5 mT; sweep time, 2 min; modulation width, 0.1 mT; amplitude, 2000; time constant, 0.3 s; and microwave power, 6.3 mW. ESR signal intensity was quantified as the peak-to-trough height of the first doublet, calculated as the sum of the absolute values of its maximum and minimum amplitudes. The obtained value was normalized to the Mn^2^⁺ marker signals recorded simultaneously at both ends of the spectrum.

### 2.4. Confocal imaging

Leaves were gently excised from the base and incubated for 30 min in culturing water containing 1% (w/v) lidocaine. After being rinsed twice, the leaves were exposed for 10 min to culturing water supplemented with 0.1% (v/v) Triton X-100. Subsequently, the samples were washed again with culturing water and incubated for 10 min with Phalloidin-iFluor 555 to visualize actin filaments. The staining solution was prepared by diluting the supplied stock solution (1,000× phalloidin conjugate in DMSO) 1,000-fold in PBS containing 1% bovine serum albumin, yielding a final DMSO concentration of 0.1% (v/v). Confocal images were acquired using an FV4000 confocal laser scanning microscope (Evident Co., Ltd., Tokyo, Japan) equipped with a 60× oil immersion objective lens (NA 1.42). Excitation and emission wavelengths were set at 561 nm and 570–620 nm, respectively.

Production of reactive oxygen species (ROS) in *E. densa* leaves was analyzed using 2', 7'-dichlorofluorescein diacetate (H_2_DCFDA). Before staining, leaves were incubated for 30 min in different pretreatment solutions. Pretreatment conditions included culturing water alone (untreated control), 10 μM VAS2870 as an inhibitor of nicotinamide adenine dinucleotide phosphate (NADPH) oxidase, 0.3 mM N-acetyl cysteine (NAC; antioxidant), 2 mM ethylene glycol-bis(*β*-aminoethyl ether)-N, N, N', N'-tetraacetic acid (EGTA; Ca^2^⁺ chelator), 0.5 mM lanthanum chloride (LaCl_3_; Ca^2^⁺ channel blocker), and 50 μM gadolinium chloride (GdCl_3_; Ca^2^⁺ channel blocker). Following pretreatment, leaves were rinsed twice with culturing water and then incubated for 10 min in 50 μM H_2_DCFDA solution containing 0.1% (v/v) DMSO. After two additional washes, the leaves were mounted onto glass slides. To induce ROS production, culture water containing 2% (w/v) lidocaine was added immediately before initiation of time-lapse imaging. The lidocaine concentration was determined based on the optimal conditions identified in the ESR experiment described above. Fluorescence imaging was performed using an FV4000 confocal laser scanning microscope equipped with a 40× dry objective lens (NA 0.95). The digital zoom factor was set to 2.0×, and image acquisition was performed at a sampling speed of 4.0 μs/pixel. H_2_DCFDA fluorescence was excited with a 488-nm laser and detected between 500 and 540 nm. Time-lapse images were collected every 2 s for a total of 101 frames, corresponding to a total recording time of 200 s. Fluorescence intensity within fixed-size regions of interest (ROIs) was quantified using cellSens software (Evident Co., Ltd., Tokyo, Japan). For each experiment, one field of view was recorded from each of three different leaves. Mean fluorescence intensities were calculated from three ROIs per field (total *n =* 9) and plotted over time. To assess the effects of pretreatment on lidocaine-induced ROS production, fluorescence intensities at 60, 120, and 180 s were compared.

### 2.5. Statistics

Statistical analyses were performed using JMP Pro version 18.0.2 (JMP Statistical Discovery LLC, Cary, NC, USA). Cytoplasmic streaming velocities were compared using Student’s *t*-test, with statistical significance defined as *p* < 0.05. In experiments evaluating recovery of cytoplasmic streaming velocity, differences among the three experimental phases were analyzed using one-way ANOVA followed by the Tukey-Kramer test for multiple comparisons (*p* < 0.05). The Tukey HSD test was used to compare normalized ESR signal intensities (*p* < 0.05). For time-course quantification of ROS using H_2_DCFDA, a Student’s *t*-test was performed at each time point, with significance defined as *p* < 0.05 (*n* = 9). To evaluate the effects of pretreatment in the lidocaine-treated group, pairwise comparisons were performed using Student’s *t*-test at representative time points (60, 120, and 180 s). Groups indicated by different letters were considered significantly different from the untreated group (*p* < 0.05).

### 2.6. Chemicals

Lidocaine hydrochloride was purchased from Combi-Blocks (San Diego, CA, USA). Ethanol, dimethyl sulfoxide (DMSO), lanthanum chloride (LaCl_3_), gadolinium chloride (GdCl_3_), N-acetyl-L-cysteine (NAC), and L-(+)-ascorbic acid (AsA) were obtained from FUJIFILM Wako Pure Chemical Corporation (Osaka, Japan). VAS2870 was purchased from Sigma-Aldrich (St. Louis, MO, USA). Ethylene glycol-bis(*β*-aminoethyl ether)-N, N, N', N'-tetraacetic acid (EGTA) was obtained from Tokyo Chemical Industry Co., Ltd. (TCI; Tokyo, Japan). *α*-(4-Pyridyl-1-oxide)-N-tert-butylnitrone (POBN) was purchased from Enzo Life Sciences, Inc. (Farmingdale, NY, USA). Triton X-100 was obtained from Nacalai Tesque, Inc. (Kyoto, Japan). Phalloidin-iFluor 555 was obtained from Cayman Chemical Company (Ann Arbor, MI, USA), and H_2_DCFDA was purchased from Invitrogen (Thermo Fisher Scientific, Waltham, MA, USA).

## 3. Results

### 3.1. Lidocaine reversibly decelerated cytoplasmic streaming

**Figure 1.**
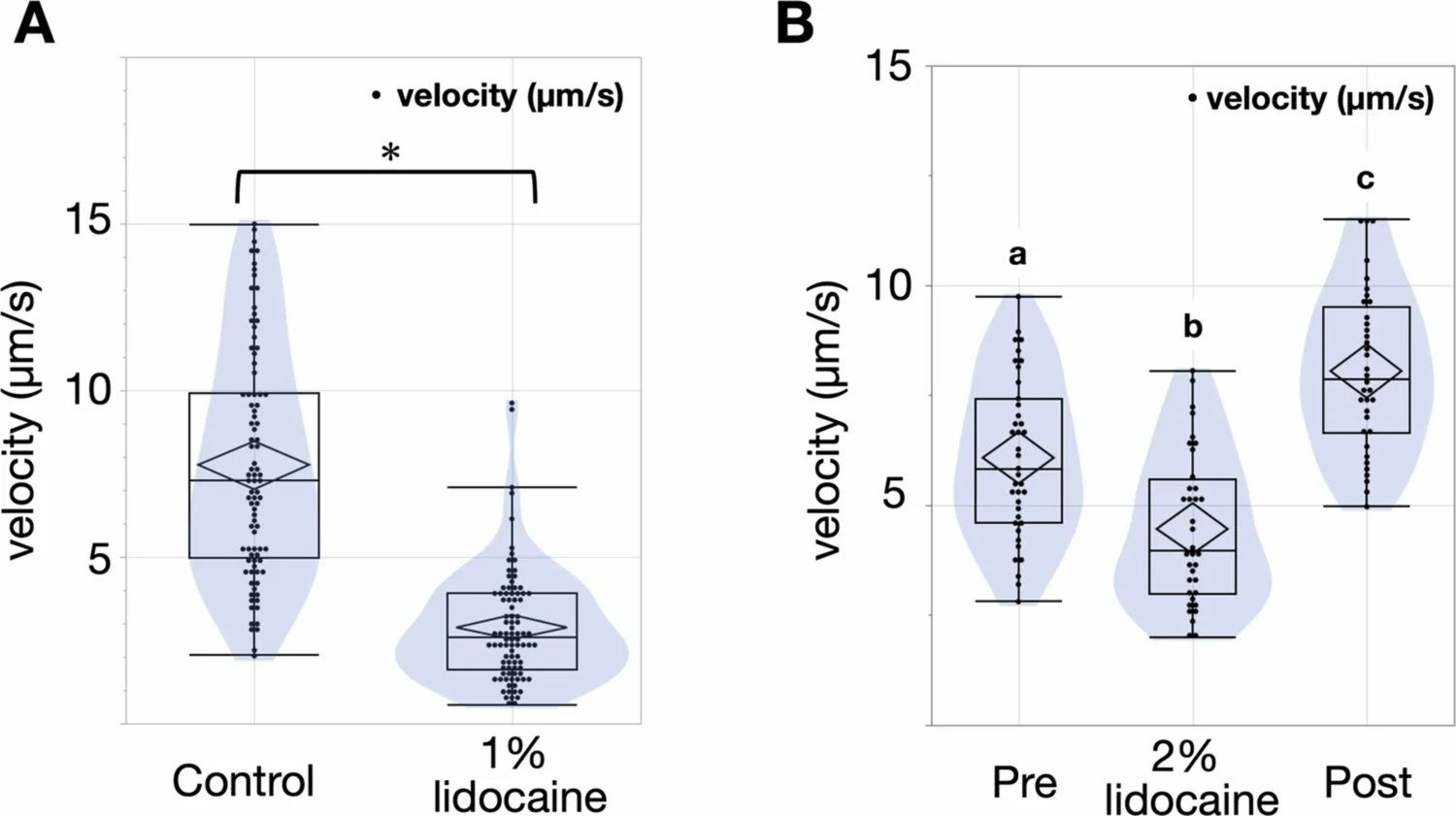
Reduction of cytoplasmic streaming velocity by lidocaine treatment and subsequent recovery. (A) In the control group, cytoplasmic streaming velocity was 7.75 ± 0.26 μm/s (mean ± SE, *n =* 89), whereas treatment with 1% lidocaine reduced the velocity to 2.90 ± 0.26 μm/s (mean ± SE, *n =* 90). In the box plots, the lower and upper boundaries of the boxes indicate the first and third quartiles, respectively, while the central horizontal line represents the median value. In the mean diamonds, the upper and lower endpoints correspond to the 95% confidence intervals, and the central horizontal line indicates the mean value. Statistical significance was evaluated using Student’s *t*-test (*p < 0.05). (B) During the pretreatment phase, cytoplasmic streaming velocity was 6.07 ± 0.28 μm/s, which decreased to 4.46 ± 0.30 μm/s during treatment with 2% lidocaine and subsequently increased to 8.06 ± 0.30 μm/s during the post-treatment phase. Following washout of lidocaine, the recovered streaming velocity significantly exceeded the initial pretreatment level (Tukey-Kramer test, *p* < 0.05; *n =* 39, 36, and 36, respectively).

To determine the cytoplasmic streaming velocity in *Egeria densa*, chloroplast movement was tracked and measured. The streaming velocity in the control group was 7.75 ± 0.26 μm/s (mean ± standard error [SE]), which was consistent with previously reported values for this species. Treatment with 1% lidocaine significantly reduced the streaming velocity to 2.90 ± 0.26 μm/s (*p* < 0.05; *n =* 89, 90; Figure 1A).

In the recovery experiment, the streaming velocity during the pretreatment phase was 6.07 ± 0.28 μm/s (mean ± SE). Treatment with 2% lidocaine reduced the velocity to 4.46 ± 0.30 μm/s, whereas the velocity increased to 8.06 ± 0.30 μm/s during the post-treatment phase. This value was significantly higher than that observed during the pretreatment phase (*p* < 0.05; *n =* 39, 36, 36; Figure 1B).

### 3.2. Local anesthetic disrupted the actin cytoskeleton

**Figure 2.**
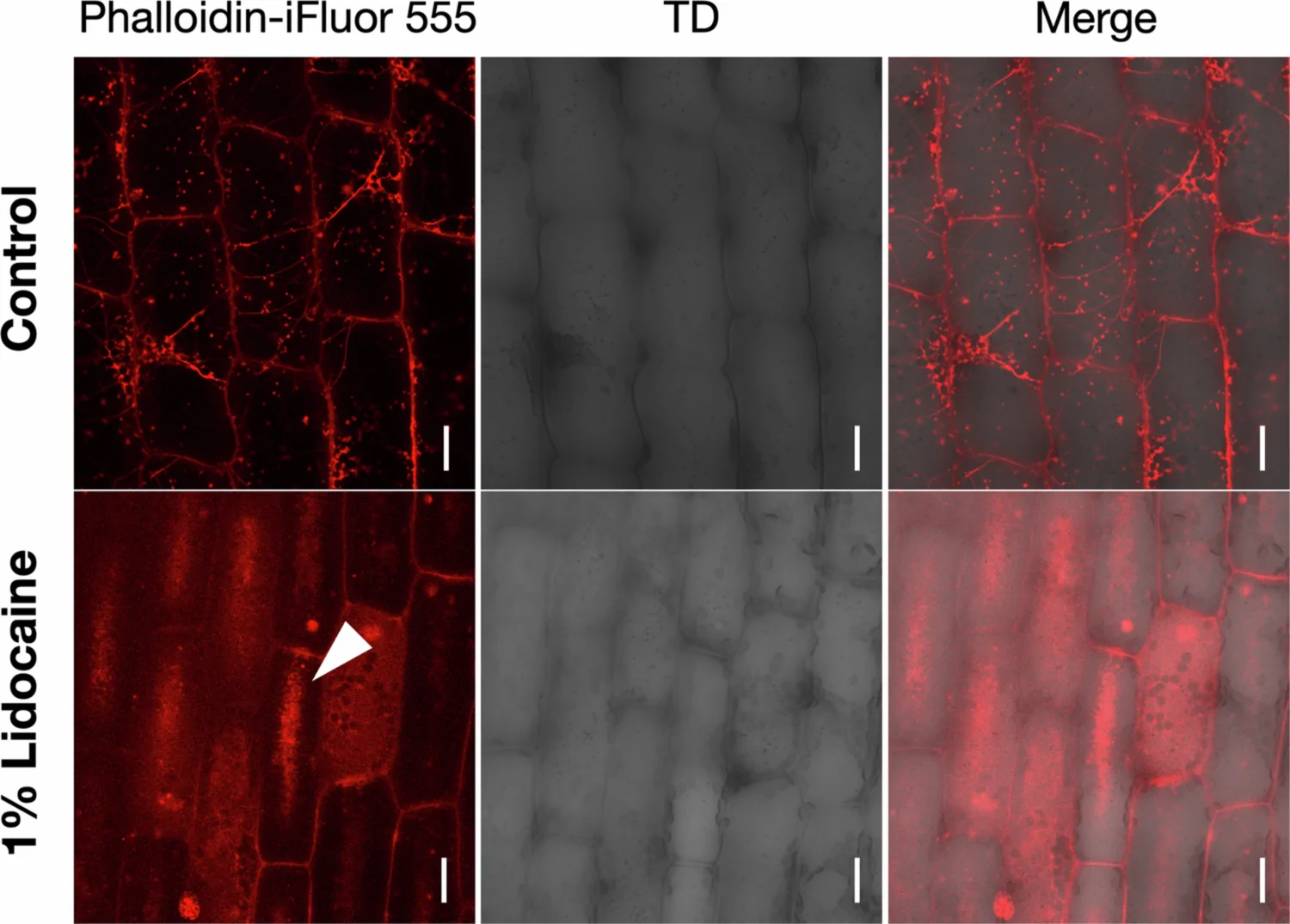
Visualization of actin filaments in *Egeria densa* cells using Phalloidin-iFluor 555. A distinct network of filamentous actin was observed in the control group, whereas lidocaine treatment caused the actin filaments to disintegrate into granular structures. The arrowhead indicates disrupted actin filaments. TD, transmitted light image. Scale bar = 20 μm.

To visualize actin filaments in *E. densa*, leaves were stained with Phalloidin-iFluor 555, a fluorescently labeled phalloidin reagent (Figure 2). Pretreatment with 0.1% Triton X-100 was required to facilitate effective penetration of the reagent into the cells. In untreated control leaves, distinct filamentous actin fibers were clearly observed, and chloroplasts were positioned adjacent to these filaments. In contrast, treatment with 1% lidocaine disrupted the actin cytoskeleton into fine granular structures.

### 3.3. Lidocaine-induced ROS generation

**Figure 3.**
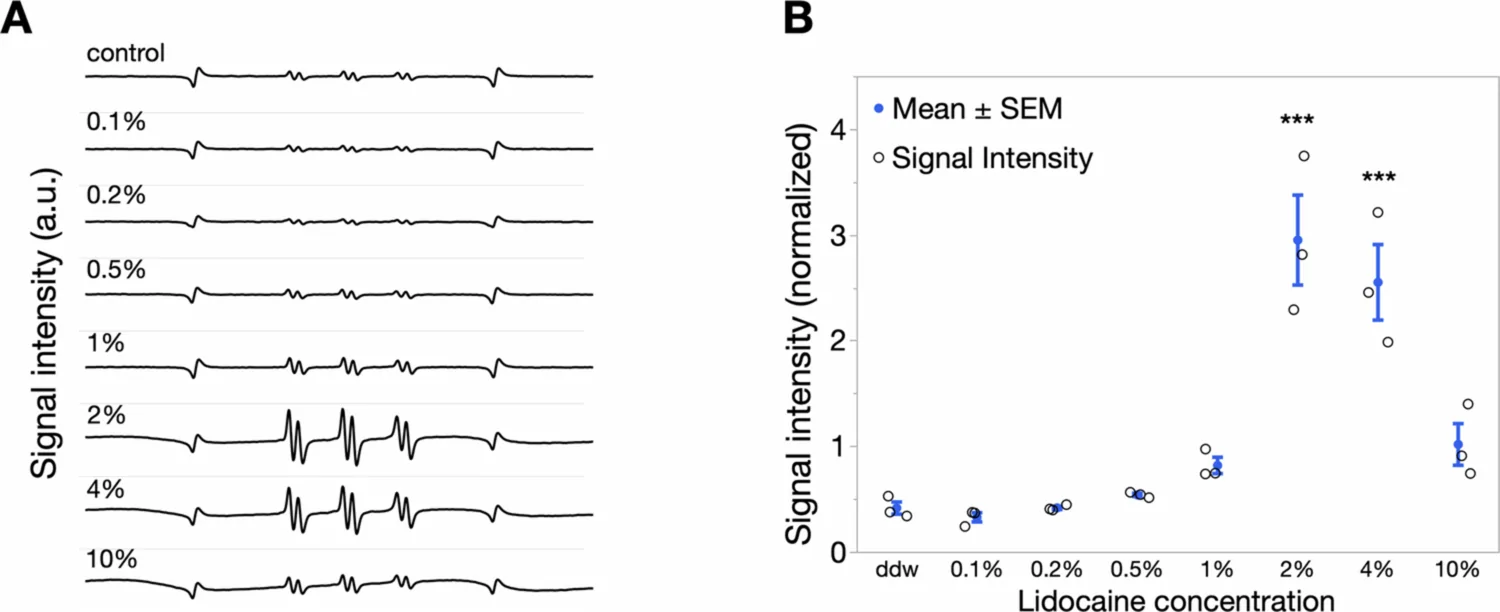
Detection and quantification of ROS production in *Egeria densa* leaves treated with lidocaine. (A) ESR spectra of radical adducts detected in *E. densa* leaves after 1 h of treatment with various concentrations of lidocaine. The two outer signals represent the Mn^2+^ marker, while the three central signals correspond to POBN. ESR signals are presented as the mean values of three replicates. (B) Signal intensities normalized to the Mn^2^⁺ marker signal, calculated from the peak-to-trough heights of the ESR spectra. Individual data points (*n* = 3) are shown with means ± SE. Asterisks indicate significant differences from the control group (Tukey HSD; ***p < 0.001).

We investigated whether LAs induce ROS production in whole leaves of *Egeria densa* using electron spin resonance (ESR). ESR enables quantitative evaluation of ROS generation. The spin-trapping reagent POBN is suitable for detecting carbon-centered and lipid-derived radicals in biological samples.^18^ Among ROS detection methods, the POBN-EtOH system enables detection of extracellular hydroxyl radical generation in plant cells.^19^ To evaluate the dose-response relationship, experiments were conducted using lidocaine concentrations ranging from 0.1% to 10% (Figure 3A). The mean normalized signal intensities (± SE) for the lidocaine-treated groups were 0.327 ± 0.043 (0.1% lidocaine), 0.416 ± 0.015 (0.2%), 0.539 ± 0.015 (0.5%), 0.818 ± 0.077 (1%), 2.949 ± 0.425 (2%), 2.550 ± 0.357 (4%), and 1.016 ± 0.197 (10%), whereas the normalized signal intensity in the control group was 0.414 ± 0.058 (Figure 3B). Normalized ESR signal intensity increased in a dose-dependent manner up to a concentration of 2% and declined at higher concentrations. Normalized signal intensities in the 2% and 4% groups were significantly higher than those in the control group (*p* < 0.001, Tukey HSD). Three biological replicates were performed for each experiment.

### 3.4. Time-lapse confocal imaging of cellular ROS production

**Figure 4.**
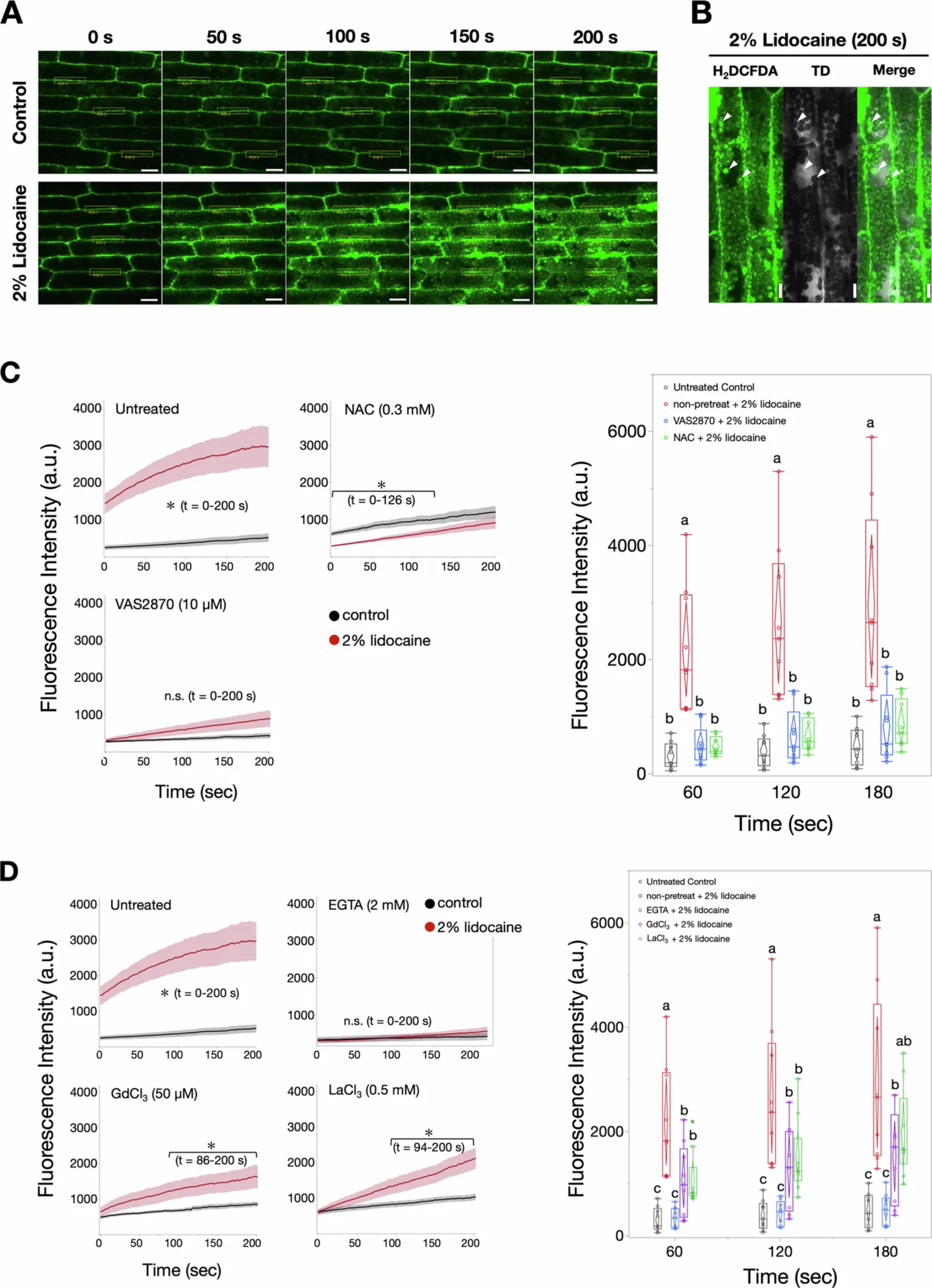
Quantification of lidocaine-induced ROS production in *Egeria densa* leaves using H_2_DCFDA. (A) Time-lapse imaging of lidocaine-induced ROS production. Representative images acquired every 50 s are shown (bar = 20 μm). (B) Lidocaine treatment increased fluorescence intensity around the cell membrane and within the central regions of chloroplasts at 200 s (bar = 10 μm). (C) Inhibitory effects of an NADPH oxidase inhibitor and antioxidant on lidocaine-induced ROS production. Time-course changes in fluorescence intensity, reflecting ROS production, were measured in leaves pretreated with the NADPH oxidase inhibitor VAS2870 or the antioxidant NAC before lidocaine treatment. Individual data points (*n* = 9) are shown with means ± SE. Student’s *t*-tests were performed to compare the control and lidocaine-treated groups at each time point (*p* < 0.05). Fluorescence intensities at representative time points (60, 120, and 180 s) are compared; box plots indicate the maximum and minimum values, whereas diamonds represent the means with 95% confidence intervals. Pretreatment with VAS2870 or NAC significantly suppressed the lidocaine-induced increase in fluorescence relative to the untreated group. Statistical significance was determined using Student’s *t*-test for pairwise comparisons at each time point, and different letters indicate significant differences (*p* < 0.05). (D) Role of Ca^2^⁺ in ROS production. Leaves were pretreated with a Ca^2^⁺ chelator (EGTA) or Ca^2^⁺ channel blockers (GdCl_3_ and LaCl_3_) before lidocaine treatment. Significant differences were observed at the indicated time points. Fluorescence intensities at representative time points (60, 120, and 180 s) are compared. Pretreatment with EGTA, GdCl_3_, and LaCl_3_ significantly suppressed the lidocaine-induced increase in fluorescence relative to the untreated group. Statistical analyses were performed as described in Figure 4C.

Subsequently, ROS production was observed at the cellular level using confocal laser scanning microscopy. H_2_DCFDA was used because its diacetate groups render the molecule hydrophobic, thereby facilitating membrane permeation. Once inside the cell, intracellular esterases deacetylate the acetyl groups. The resulting H_2_DCF accumulates intracellularly and emits fluorescence upon reacting with ROS. In E. *densa* leaves treated with 2% lidocaine, increased fluorescence intensity was observed around the cell membrane and within the central regions of chloroplasts (Figure 4A, B). Time-lapse analysis focused on the cell periphery revealed that fluorescence intensity significantly increased in the lidocaine-treated group relative to the control group. Although lidocaine induced a significant increase in fluorescence in the absence of inhibitors, this effect was suppressed in the VAS2870-pretreated group (NADPH oxidase inhibitor), and no significant difference was observed between the control and lidocaine-treated cells under this condition (Figure 4C). When pretreated with the antioxidant NAC, fluorescence intensity in the lidocaine-treated group was significantly lower than that in the non-pretreated lidocaine-treated group. Furthermore, among NAC-pretreated samples, fluorescence intensity in lidocaine-treated cells was lower than that in the control group, particularly during the interval from 0 to 126 s (*p* < 0.05; Figure 4C). Pretreatment with either VAS2870 or NAC significantly suppressed the lidocaine-induced increase in fluorescence intensity at 60, 120, and 180 s relative to the untreated group (*p* < 0.05). To facilitate direct comparison with the untreated control, the control group was included in the box plots. At 60, 120, and 180 s, fluorescence intensity in both the VAS2870- and NAC-pretreated groups was significantly lower than that in the non-pretreated lidocaine-treated group, whereas neither group differed significantly from the untreated control group (*p* < 0.05; Figure 4C). No significant difference was observed between the VAS2870- and NAC-pretreated groups at these time points.

### 3.5. Ca^2^⁺ under lidocaine treatment

Next, to investigate whether Ca^2^⁺ influx is involved in lidocaine-induced ROS production, experiments were conducted using a Ca^2^⁺ chelator (EGTA) and ion channel blockers (i.e., GdCl_3_ and LaCl_3_). Pretreatment with EGTA, which chelates extracellular Ca^2^⁺, caused a substantial reduction in fluorescence intensity in both the control and lidocaine-treated groups. In contrast, in the presence of the calcium channel blockers GdCl_3_ and LaCl_3_, the lidocaine-treated group exhibited a time-dependent increase in fluorescence intensity relative to the control group. In the GdCl_3_ group, fluorescence intensity became significantly higher than that of the control after 86 s, whereas in the LaCl_3_ group, the increase became significant after 94 s (Figure 4D). At 60, 120, and 180 s, fluorescence intensities were significantly lower in all pretreated groups than in the untreated group, with the greatest reduction observed in the EGTA-treated group. When the untreated control group was also included in the analysis, fluorescence intensity in the EGTA-pretreated group did not differ significantly from that in the untreated control group at any of the three time points. In contrast, the GdCl_3_-pretreated group remained significantly different from both the untreated control and untreated lidocaine-treated groups at 60, 120, and 180 s. The LaCl_3_-pretreated group showed a similar pattern at 60 and 120 s, whereas at 180 s it did not differ significantly from either the GdCl_3_-pretreated group or the untreated lidocaine-treated group.

### 3.6. Attenuation of lidocaine-induced reduction in cytoplasmic streaming velocity by ROS inhibitors

**Figure 5.**
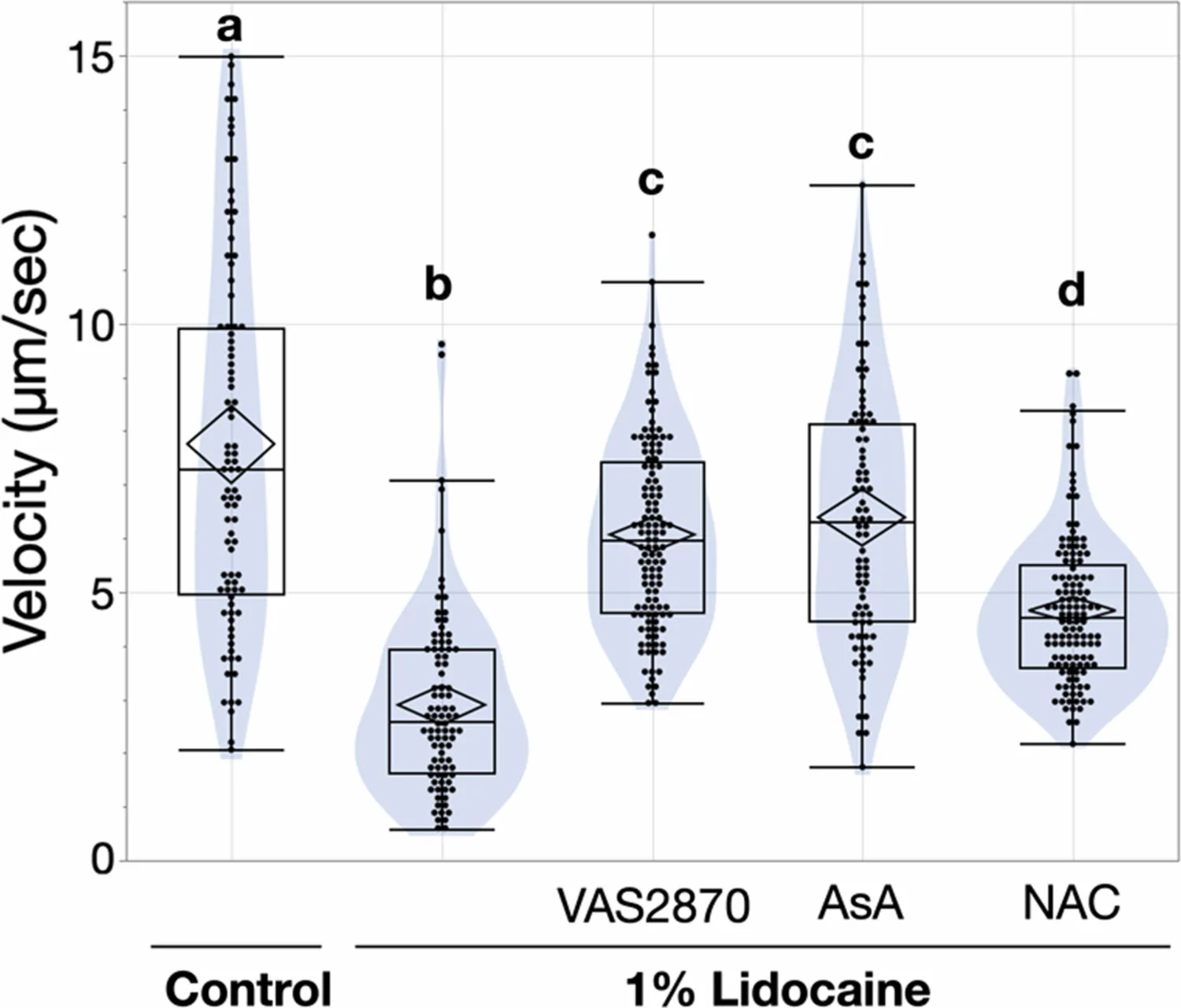
ROS inhibitors alleviate the lidocaine-induced reduction in cytoplasmic streaming velocity. Pretreatment with VAS2870, ascorbic acid (AsA), or N-acetyl cysteine (NAC) significantly attenuated the reduction in streaming velocity. Data are presented as mean ± SE. Different letters indicate significant differences (Tukey-Kramer test, *p* < 0.05). Values for each group are as follows: lidocaine-free control (7.75 ± 0.23 μm/s, *n =* 89); 1% lidocaine-only (2.90 ± 0.23 μm/s, *n =* 90); pretreatment with VAS2870 (6.06 ± 0.20 μm/s, *n =* 122), AsA (6.40 ± 0.23 μm/s, *n =* 85), and NAC (4.67 ± 0.20 μm/s, *n =* 124). Although significant prevention of the velocity reduction was observed in all pretreatment groups, these values remained significantly lower than those of the lidocaine-free control group.

We investigated whether reducing lidocaine-induced oxidative stress could prevent the reduction in cytoplasmic streaming velocity. The results showed that pretreatment with VAS2870 (an NADPH oxidase inhibitor), as well as NAC and AsA (ROS scavengers), significantly attenuated the lidocaine-induced decrease in streaming velocity (Figure 5). The mean velocity in the 1% lidocaine-only group was 2.90 ± 0.23 μm/s (*n =* 90). In contrast, the velocities in groups pretreated with VAS2870, AsA, and NAC were 6.06 ± 0.20 μm/s (*n =* 122), 6.40 ± 0.23 μm/s (*n =* 85), and 4.67 ± 0.20 μm/s (*n =* 124), respectively. Although significant prevention of the velocity reduction was observed in all pretreatment groups, these values remained lower than that of the lidocaine-free control group (7.75 ± 0.23 μm/s, *n =* 89). These findings indicate that lidocaine decreases cytoplasmic streaming velocity through ROS generation. Data are presented as mean ± SE. Statistical significance was determined using Tukey-Kramer tests (*p* < 0.05).

## 4. Discussion

### 4.1. Reversible inhibition of cytoplasmic streaming by lidocaine in *Egeria densa*

Despite their similar appearance, *Chara braunii* and *Egeria densa* are evolutionarily distant species; the former is an alga, whereas the latter is an angiosperm. LAs have been reported to inhibit cytoplasmic streaming in *Chara.* ^11^ In the present study, we observed a similar effect in *E. densa*, demonstrating that lidocaine treatment reversibly decreases cytoplasmic streaming velocity. Although LAs are known to exhibit cytotoxicity at high concentrations, we observed that the streaming velocity fully recovered and even exceeded the baseline level 1 h after lidocaine treatment. These findings indicate that cell viability is maintained following a 30-min treatment with 2% lidocaine. It should be noted that, although every effort was made to control and standardize the experimental conditions (including maintaining a constant temperature of approx. 24 °C), baseline cytoplasmic streaming velocities in *E. densa* naturally exhibit physiological variations depending on individual plant conditions, leaf maturity, light history, and experimental batches. Such inherent variations likely account for the slight baseline differences observed between independent experimental sets.

### 4.2. Actomyosin system involvement

The core machinery underlying cytoplasmic streaming consists of actin filaments that function as intracellular tracks, myosin-dependent motility, and ATP as the driving energy source. Mechanical stimulation, including physical contact, induces action potentials in the plasma membrane. These electrical responses are accompanied by rapid influx of Ca^2^⁺ into the cytoplasm from extracellular regions or intracellular stores.^20^ ^,^ ^21^ In the alga *Chara*, experimental studies demonstrated that action potential-induced Ca^2^⁺ influx rapidly terminates cytoplasmic streaming.^22^ This inhibitory mechanism, which effectively acts as a “brake” on cytoplasmic streaming, has been termed “mechanically-induced excitation-cessation coupling” (E-C coupling).^23^ Such transient cessation of streaming is considered part of a plant defense response that physically restricts pathogen spread and prevents leakage of cytoplasmic contents. Consistent with this interpretation, exposure to chloroform vapor suppresses mechanically induced cessation of cytoplasmic streaming in *Chara.* ^24^

Previous studies further demonstrated that Ca^2^⁺ influx promotes dissociation of calmodulin (CaM) from the neck domain of myosin XI. This conformational alteration, particularly shortening of the neck region, markedly decreases step size, sliding velocity, and ATPase activity of the motor protein.^25^ Elevated intracellular Ca^2^⁺ concentrations also destabilize the actin bundles that serve as tracks for myosin movement. Through activation of the Ca^2^⁺ sensor CaM and actin-binding proteins such as villin, increased intracellular Ca^2^⁺ promotes fragmentation of actin bundles.^26-28^ Consequently, the reduction in cytoplasmic streaming velocity may arise from both dysfunction of the myosin motor and disruption of the actin filament network required for organelle transport. In the present study, fluorescent phalloidin staining enabled visualization of actin filaments in *E. densa*, revealing that lidocaine treatment disrupted normal actin filament organization. However, evaluating whether pretreatment with calcium channel blockers (GdCl_3_ and LaCl_3_) or EGTA could prevent the lidocaine-induced reduction in streaming velocity proved unsuccessful; treatment with these calcium-modulating agents alone led to the cessation of streaming and chloroplast aggregation, despite extensive optimization. A limitation of phalloidin staining is its irreversible binding to F-actin, which precludes tracking live-cell actin dynamics or recovery after lidocaine washout. Additionally, whether elevated intracellular Ca^2^⁺ directly impairs myosin motility under these conditions remains unclear and requires further investigation.

LAs may additionally suppress ATP production. Previous studies have shown that, in plants, LAs disrupt photophosphorylation within thylakoid membranes and depolarize the mitochondrial inner membrane.^29-32^ In mammalian mitochondria, these effects reportedly involve inhibition of electron transport chain complexes I and III, leading to enhanced ROS production.^33^ Such mitochondrial and chloroplast dysfunction would be expected to reduce intracellular ATP availability. Supporting this possibility, Sylvain-Bonfanti et al. reported that lidocaine treatment significantly decreased intracellular ATP levels in cultured rice cells.^16^ Therefore, the reduction in cytoplasmic streaming velocity observed in this study is likely mediated by multiple interconnected mechanisms. Lidocaine appears to impair not only actomyosin-based motility but also ATP generation, which is essential for sustaining intracellular streaming.

Interestingly, following anesthetic washout, we observed an overshoot phenomenon in which cytoplasmic streaming velocity transiently exceeded the control level. One possible explanation is rapid consumption of ATP or sugar reserves accumulated during anesthesia. Alternatively, prolonged ATP depletion during lidocaine treatment may trigger compensatory metabolic responses that transiently enhance streaming activity after recovery. Additional studies will be necessary to determine the mechanisms responsible for this rebound effect.

### 4.3. ROS-induced reduction in cytoplasmic streaming velocity

It is well established that anesthetics induce ROS generation in plants.^15-17^ ROS are primarily produced by integral plasma membrane proteins, including NADPH oxidases, which are also referred to as respiratory burst oxidase homologs.^34^ ^,^ ^35^ Consistent with these previous reports, we observed using H_2_DCFDA that lidocaine treatment similarly triggered ROS production in *E. densa* leaves. High levels of ROS were detected around the cell membrane and within the central regions of chloroplasts. Because this local anesthetic-induced ROS production was strongly inhibited by VAS2870, an NADPH oxidase inhibitor, these findings suggest that ROS production under lidocaine treatment is mediated by NADPH oxidases. Some plant NADPH oxidases possess intracellular EF-hand domains that bind Ca^2^⁺.^36^ ^,^ ^37^ Because ROS production was inhibited by both a chelator (EGTA) and calcium channel blockers (GdCl_3_ and LaCl_3_), LAs likely trigger ROS production through induction of extracellular Ca^2^⁺ influx. Suppression of ROS production by the antioxidant NAC may be attributable to attenuation of the ROS-induced ROS release (RIRR) cycle caused by lidocaine-induced ROS.^38^ ^,^ ^39^

Regarding ROS generation in chloroplasts, mechanisms other than NADPH oxidases are likely involved. Inhibition of the electron transport chain by LAs may induce electron leakage from chloroplast photosystems, thereby generating superoxide through electron transfer to oxygen. Previous studies have demonstrated that chloroplast-derived ROS promote actin depolymerization.^40^ Because chloroplasts are positioned close to actin filaments in *E. densa*, chloroplast-derived ROS may contribute to reorganization of the actin cytoskeleton. We propose that restoration of streaming velocity by VAS2870, ascorbic acid, and NAC resulted from attenuation of the RIRR loop. This reduction in overall ROS levels may have partially preserved electron transport chain activity, photosystem function, and actin dynamics. Furthermore, it is worth noting that normalized ESR signal intensity exhibited an inverted U-shaped dose response, peaking at 2% lidocaine and declining at higher concentrations. While our recovery assays indicated that lidocaine-induced effects remain largely reversible up to 2%, exposure to concentrations exceeding 2% likely imposes excessive stress on the cells, which may impair membrane integrity and cellular viability, thereby suppressing the metabolic machinery required for ROS generation—a phenomenon reminiscent of a hormetic-like stress response.

In summary, this study visually demonstrated that the reduction in cytoplasmic streaming velocity induced by LAs is associated with disruption of the actomyosin system, likely caused by rapid Ca^2^⁺ influx and breakdown of actin bundles accompanied by ROS overproduction through RIRR. Importantly, these responses were reversible and did not impair cell viability. We also observed a “rebound effect,” in which cytoplasmic streaming velocity transiently exceeded the baseline level after anesthetic removal. These findings suggest that LAs influence the maintenance mechanisms underlying metabolic homeostasis.

## 5. Conclusion

Our findings indicate that lidocaine inhibits cytoplasmic streaming in *Egeria densa* through the induction of ROS production, Ca^2+^ influx, and disruption of the actomyosin system. These responses are reversible and do not impair cell viability; however, the subsequent “rebound effect” suggests that LAs transiently interfere with the fundamental maintenance mechanisms of metabolic homeostasis.
